# The Diseases of the Nervous System

**Published:** 1899-10

**Authors:** 


The Diseases of the Nervous System. A Text-Book for Physicians and Students. By Dr. Ludwig Hirt, Professor of the University of Breslau. Second American edition, revised from the second German edition by August Hoch, M. D., assisted by Frank R. Smith, A. M. (Cantab.), M. D., Assistant Physicians to the Johns Hopkins Hospital. With an Introduction by William Osler, M. D., F. R. C. P., Professor of Medicine in the Johns Hopkins University. Octavo, with 178 illustrations. Sold only by subscription. New York: D. Appleton & Co. 1899.

The distinct value to the American physician of this work lies in the fact that he sees the suggestion presented from a different point of view.

American neurologists have their theories and have proclaimed them so long that one is almost inclined to accept them as undisputable facts.

Hirt's work is a representative presentation of German neurology. In the description of diseases we find the work clear and careful.

In the acceptation of theories, such as the cause of acromegaly, being enlargement of the hypophysis. He accepts none of the theories advanced as to the pathology of chorea. Of anterior poliomyelitis he says it is possible that infectious influences will at some time be proved to be the cause of the disease; for the present, however, this is nothing more than a hypothesis, which has not gained any firmer ground from a report of Cordier of an epidemic of the disease. Throughout the work we note the care and thoroughness of the author in weighing evidence. His extreme caution in accepting only the proven makes his volume a landmark of the present boundaries of our knowledge in this field. J. W. P.

